# The Added Value of In-Hospital Tracking of the Efficacy of Decongestion Therapy and Prognostic Value of a Wearable Thoracic Impedance Sensor in Acutely Decompensated Heart Failure With Volume Overload: Prospective Cohort Study

**DOI:** 10.2196/12141

**Published:** 2020-03-18

**Authors:** Christophe J P Smeets, Seulki Lee, Willemijn Groenendaal, Gabriel Squillace, Julie Vranken, Hélène De Cannière, Chris Van Hoof, Lars Grieten, Wilfried Mullens, Petra Nijst, Pieter M Vandervoort

**Affiliations:** 1 Mobile Health Unit Faculty of Medicine and Life Sciences Hasselt University Hasselt Belgium; 2 Connected Health Solutions Holst Centre/Interuniversity Microelectronics Center The Netherlands Eindhoven Netherlands; 3 Future Health Department Ziekenhuis Oost-Limburg Genk Belgium; 4 Interuniversity Microelectronics Center Heverlee Belgium; 5 Department of Cardiology Ziekenhuis Oost-Limburg Genk Belgium

**Keywords:** congestive heart failure, electric impedance, prognosis

## Abstract

**Background:**

Incomplete relief of congestion in acute decompensated heart failure (HF) is related to poor outcomes. However, congestion can be difficult to evaluate, stressing the urgent need for new objective approaches. Due to its inverse correlation with tissue hydration, continuous bioimpedance monitoring might be an effective method for serial fluid status assessments.

**Objective:**

This study aimed to determine whether in-hospital bioimpedance monitoring can be used to track fluid changes (ie, the efficacy of decongestion therapy) and the relationships between bioimpedance changes and HF hospitalization and all-cause mortality.

**Methods:**

A wearable bioimpedance monitoring device was used for thoracic impedance measurements. Thirty-six patients with signs of acute decompensated HF and volume overload were included. Changes in the resistance at 80 kHz (R_80kHz_) were analyzed, with fluid balance (fluid in/out) used as a reference. Patients were divided into two groups depending on the change in R_80kHz_ during hospitalization: increase in R_80kHz_ or decrease in R_80kHz_. Clinical outcomes in terms of HF rehospitalization and all-cause mortality were studied at 30 days and 1 year of follow-up.

**Results:**

During hospitalization, R_80kHz_ increased for 24 patients, and decreased for 12 patients. For the total study sample, a moderate negative correlation was found between changes in fluid balance (in/out) and relative changes in R_80kHz_ during hospitalization (rs=-0.51, *P*<.001). Clinical outcomes at both 30 days and 1 year of follow-up were significantly better for patients with an increase in R_80kHz_. At 1 year of follow-up, 88% (21/24) of patients with an increase in R_80kHz_ were free from all-cause mortality, compared with 50% (6/12) of patients with a decrease in R_80kHz_ (*P*=.01); 75% (18/24) and 25% (3/12) were free from all-cause mortality and HF hospitalization, respectively (*P*=.01). A decrease in R_80kHz_ resulted in a significant hazard ratio of 4.96 (95% CI 1.82-14.37, *P*=.003) on the composite endpoint.

**Conclusions:**

The wearable bioimpedance device was able to track changes in fluid status during hospitalization and is a convenient method to assess the efficacy of decongestion therapy during hospitalization. Patients who do not show an improvement in thoracic impedance tend to have worse clinical outcomes, indicating the potential use of thoracic impedance as a prognostic parameter.

## Introduction

Heart failure (HF) is a major and increasing public health problem worldwide and is characterized by frequent (re)hospitalizations that are mainly caused by congestion [1,2]. Congestion is related to water and sodium retention and is defined as a high left ventricular end-diastolic pressure (ie, pressure overload) followed by signs and symptoms such as dyspnea, rales, and edema (ie, volume overload) [3,4]. At present, a high dose of intravenously administered loop diuretics is the most widely used and effective therapy for fluid removal [4]. Accurately assessing a patient’s congestion status and treatment efficacy remains difficult and is mainly done by physical examination (ie, dyspnea, orthopnea, edema) or radiographic signs on chest X-ray (ie, interstitial edema, pleural effusion). Unfortunately, physical examination results and radiographic signs have poor sensitivity and predictive value [5,6]. The current gold standard to assess pressure overload is measuring right atrial and pulmonary capillary wedge pressure via cardiac catheterization [7]. However, its invasive nature limits its routine use in daily practice. Guidelines or specific criteria to define treatment efficacy and discharge readiness of patients presenting with acute decompensated HF are vague or missing. Consequently, 30% of patients still have symptoms of congestion on discharge, which negatively influences their prognosis [8].

In recent years, thoracic impedance measurements, provided by implantable (ie, OptiVol and CorVue) or external devices, have been investigated as a tool to assess fluid status and detect volume overload [9-11]. Bioimpedance is an electrical parameter that represents the resistance opposing an electrical current passing through the body. Since blood and fluids have lower resistance to an electrical current than thoracic tissue, it is theoretically possible to measure thoracic fluid changes. An inverse correlation exists between bioimpedance and the amount of body fluid [11-13]. Several invasive and portable devices can measure bioimpedance. However, due to the invasive character of implantable devices, they are only used for a subset of eligible patients. Portable devices, such as the body composition monitor from Fresenius (Bad Homburg vor der Höhe, Germany) or the Bioscan 920-II device from Maltron (Essex, United Kingdom), are bulky and can only be applied by trained medical staff. The Edema Guard Monitor from CardioSet Medical (Bnei Brak, Israel) is a portable device that can be applied in the home environment. This device can be used to predict cardiogenic pulmonary edema, but only until 60 minutes before the appearance of clinical signs, and it can prevent hospitalizations for acute HF [14,15]. Non-invasive wearable devices for bioimpedance recordings provide an interesting alternative since they enable longitudinal monitoring and trend analysis in a comfortable way [12,13,16,17]. There exists only a handful of these devices: the Cova necklace from toSense (La Jolla, CA) [18], the AVIVO Mobile Patient Management System from Corventis (San Jose, CA) [19,20], and the wearable bioimpedance vest from Philips (Andover, MA) [13,17]. The Philips vest showed a strong correlation between bioimpedance and daily weight changes, and has been shown to be able to track recompensation during therapy for acute congestive HF [13]. Unfortunately, the form of the vest limits in-hospital use and is less convenient for continuous, long-term, in-home monitoring. In an article published 2 years later, the researchers suggested that wearable bioimpedance systems such as the vest could provide value beyond measuring clinical improvement and provide a prognostic assessment of patients admitted for HF [17]. However, more evidence on the use of bioimpedance to track fluid changes and the relationship with clinical outcome is needed to support the translation of this approach into clinical practice. In a previous study, we provided initial results on the feasibility of the wearable bioimpedance sensor and the correlations with clinical reference measures [12].

In the current study, we aimed to determine whether bioimpedance-based monitoring can be used to assess changes in a patient’s fluid status (ie, the efficacy of decongestion therapy during hospitalization) and the relationships with both HF hospitalization and all-cause mortality. The research questions were *“Can a wearable bioimpedance sensor be used to measure changes in fluid status as a measure of the efficacy of decongestion therapy in patients hospitalized for acute decompensated HF?”* and *“Is there a relationship between changes in thoracic bioimpedance during hospitalization for acute decompensated HF and clinical outcome in terms of HF hospitalization and all-cause mortality?”*

## Methods

### Study Design

This was a prospective cohort study of patients admitted to a single tertiary care center (Ziekenhuis Oost-Limburg, Genk, Belgium). Consecutive patients admitted with signs of acute decompensated HF, for which diuretic therapy was started, were included. Diuretic therapy was initiated according to standard clinical practice. The initiation and continuation of the diuretic therapy were not dictated by the study protocol but were carried out according to standard care. Besides bioimpedance measurements, no additional tests nor treatments were performed beyond those of standard practice (eg, fluid balance, chest X-ray, echocardiographic examination). Bioimpedance measurement results were blinded for the treating physician. Patients were divided into two groups according to the change in bioimpedance during hospitalization and were clinically followed for 12 months. Clinical outcome measures were assessed at 30 days and 1 year of follow-up and included all-cause mortality, HF hospitalization, and the composite of all-cause mortality and HF hospitalization. All participants provided written, informed consent. The study complied with the Declaration of Helsinki, and the study protocol was approved by the local committee on human research.

### Study Population

Patients admitted to the emergency room with signs or symptoms of acute decompensated HF with volume overload, assessed by a dedicated HF specialist, were approached as soon as possible after triage. Symptoms of congestion were defined as pitting edema, worsening in shortness of breath or orthopnea, paroxysmal nocturnal dyspnea, wheezes, rales, or signs of congestion on chest X-ray such as the presence of pulmonary venous congestion, vascular redistribution, Kerley B lines, or blunted costophrenic angles. Patients were included only when the anticipated date of discharge was >48 hours after study screening (as estimated by the dedicated HF specialist). This study inclusion criterion was based on previous research in the field of bioimpedance monitoring in HF [11].

### Wearable Bioimpedance Monitor

**Figure 1:**
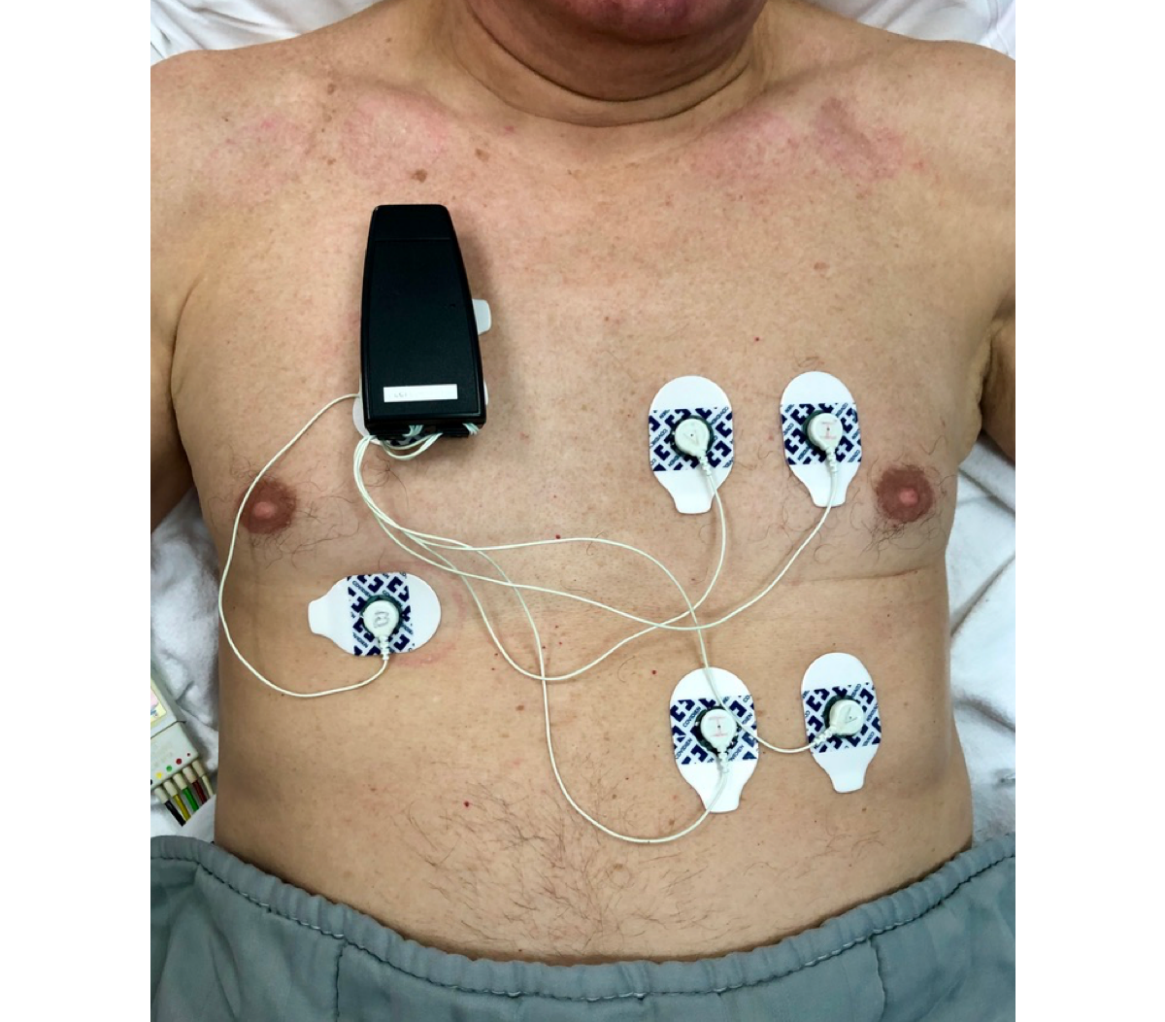
Positioning of the wearable, multi-parametric bioimpedance monitoring device from imec the Netherlands.

A novel, wearable, multi-parametric bioimpedance monitoring device from the Interuniversity Microelectronics Center (imec) the Netherlands (Eindhoven, The Netherlands) was used for local bioimpedance measurements (Figure 1). The device measures multi-frequency bioimpedance, non-standard one-lead electrocardiogram, and accelerometer data [12,16,21]. Analysis of the one-lead electrocardiogram data was out of scope for the current study since patients were monitored using a gold-standard, bedside, vital signs monitor according to standard care. Due to the dominant resistive component of fluid changes, changes in resistance at 80kHz (R_80kHz_) were used for the analyses. At this frequency, the weighted sum of extracellular water and intracellular water resistivities is being measured. This is because the current passes through both intra- and extracellular fluid, although the proportion varies from tissue to tissue. To calculate the R value per session, only bioimpedance data recorded under the same posture and during periods of low movement intensity (as measured by the accelerometer) were selected for further analysis. Next, the median R value of the filtered data per measurement was used for analysis. Bioimpedance values are strongly dependent on patient-specific characteristics, such as body composition (ie, fat percentage, muscle percentage), the amount of body hair, and skin condition. Relative bioimpedance values were therefore used for more individualized analyses and to minimalize inter-individual variability. To do so, every measurement was divided by the baseline measurement (R_n_/R_0_; R_n_ corresponds to the resistance value at time point *n*, and R_0_ corresponds to the resistance value at baseline). Patients were divided into two groups: those with a relative increase in R_80kHz_ and those with a relative decrease in R_80kHz_ from admission to coronary care unit discharge (ie, change in impedance between the very first and very last measurements). A fixed tetrapolar electrode configuration [12] was used to reduce the influence of the electrode-skin impedance.

### Measurement Protocol for Patients with Decompensated Heart Failure

Using a fixed electrode position of the wearable device, thoracic impedance measurements were performed twice a day for about 10 minutes per measurement for at least 3 consecutive days (Figure 1). Between the consecutive measurements, the device was detached; however, whenever possible, the electrodes were left in place. A skin marker was used to mark the location of the electrodes when they were removed. To eliminate the possible influence of posture, patients were always placed in a 20-30-degree semi-Fowler’s position and were asked not to move or talk during measurements. In addition, patients’ input/output fluid balances were documented every hour as a reference measure for changes in fluid status. Fluid balance was chosen as the reference measure due to its objective nature and ease of measurement in a coronary care unit where patients are equipped with a urinal or bladder probe. Fluid balance information was available until patients moved from the coronary care unit to the low intensive care unit.

### Statistical Analysis

Continuous variables are expressed as mean (SD), if normally distributed, or as median (IQR), if not normally distributed. Normality was assessed using the Shapiro-Wilk statistic. To identify statistical differences between the two groups, the independent samples student’s *t* test and Mann-Whitney *U* test were used for normally and not normally distributed continuous variables, respectively, and the Chi-Square test was used for categorical variables. Correlation analysis between each consecutive measurement for changes in fluid balance and changes in thoracic impedance values was performed using the one-tailed Spearman correlation. Survival curves were constructed according to the Kaplan-Meier method, with the log-rank test used for comparison between the groups. Cox regression analysis with Firth's penalized likelihood correction was used to calculate hazard ratios. A multivariate Cox regression model was fitted with the following explanatory variables: baseline characteristics that were significantly different between the two groups (presence of atrial fibrillation and diuretic use), clinically relevant factors (age and left ventricular ejection fraction), and the group indicator. Next, backward model building was executed, removing the explanatory variables not significant at a 5% level. A significance level of .05 was used for all tests. Cox regression with Firth’s penalization was performed using SAS 9.4 (SAS Institute Inc, Cary, NC); all other statistical analyses were performed using SPSS release 24.0 (SPSS Inc, Chicago, IL).

## Results

### Study Sample

Thirty-six patients admitted to the cardiology ward with acute decompensated HF were included with the following characteristics: mean age 81 years (SD 8 years), left ventricular ejection fraction 45% (IQR 36-55), 14 (39%) with ischemic HF etiology. Eight patients were equipped with an implantable electronic cardiac device, of which 5 patients had a pacemaker and 3 patients had a cardiac resynchronization therapy device. The mean measurement duration was 5 days (SD 2 days).

### Bioimpedance Changes

**Figure 2:**
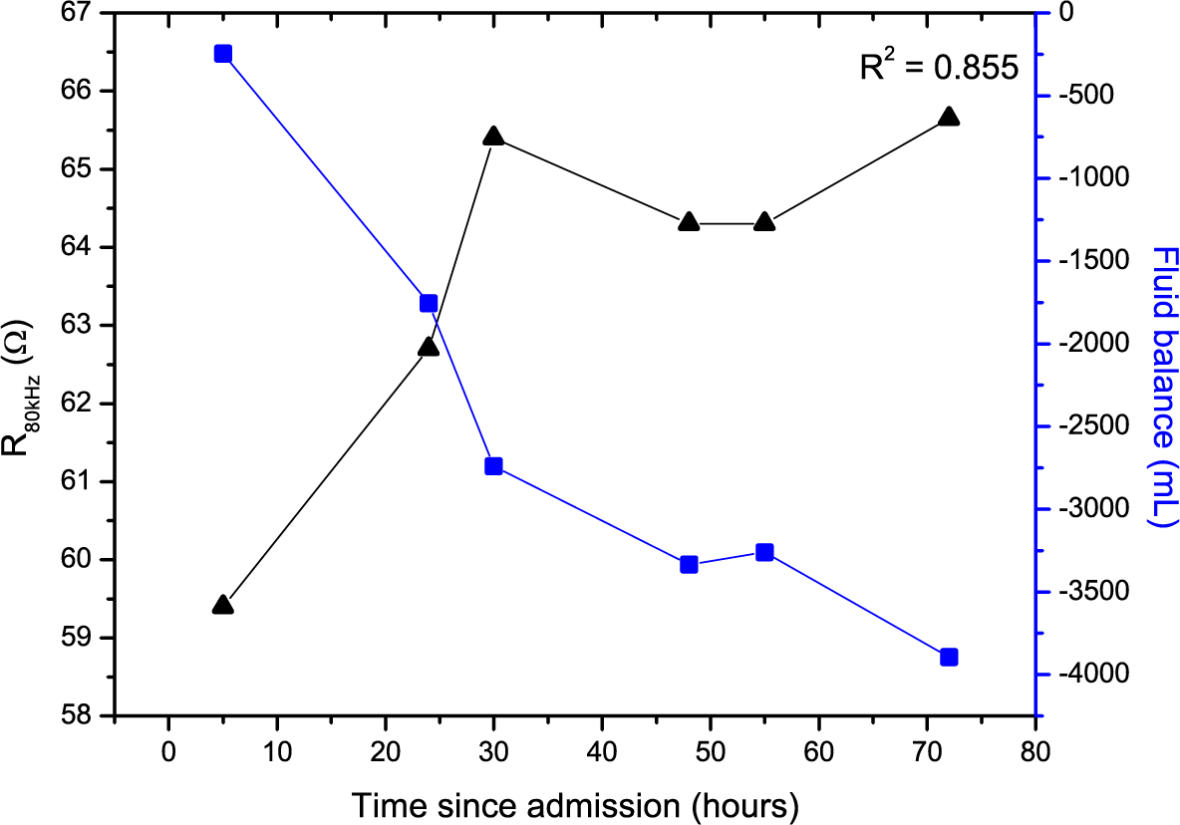
Relationship between thoracic impedance at 80 kHz (R_80kHz_; black triangles) and fluid balance (blue squares) for a representative patient admitted with combined heart failure.

**Figure 3:**
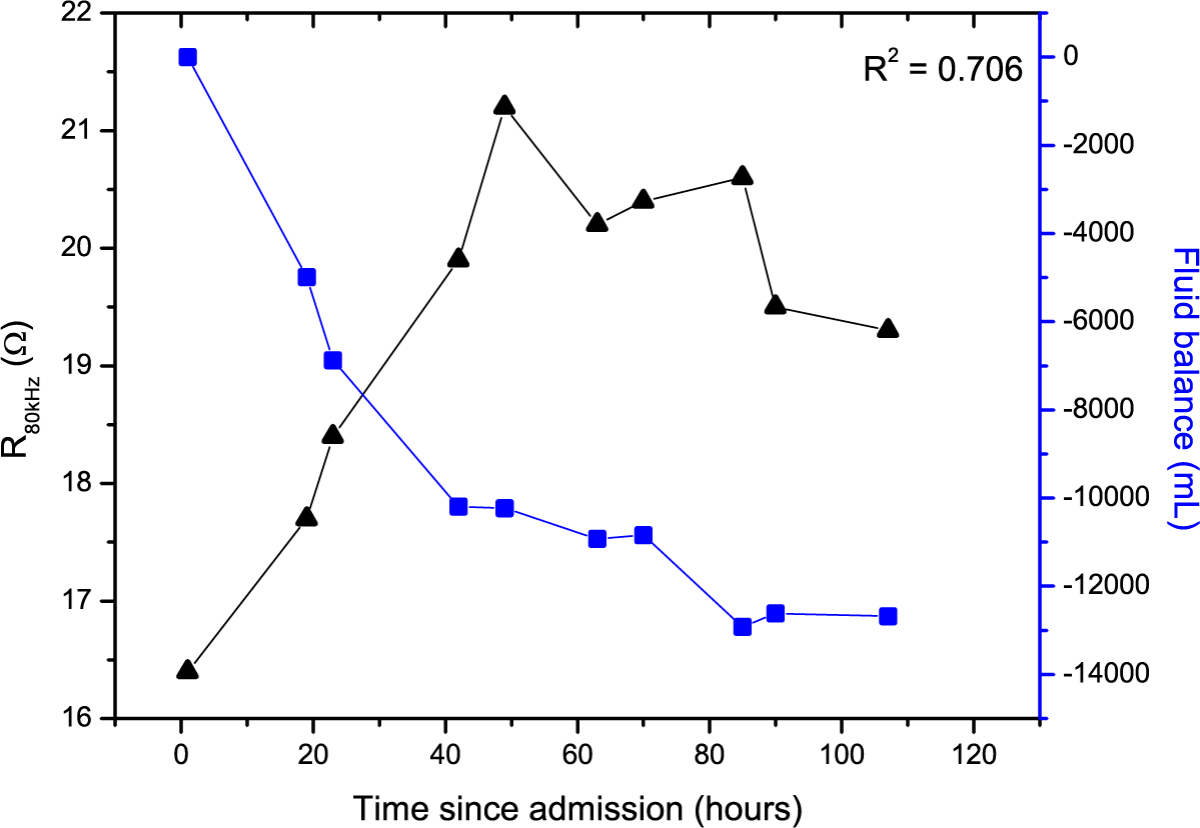
Relationship between thoracic impedance at 80 kHz (R_80kHz_; black triangles) and fluid balance (blue squares) for a representative patient admitted with isolated left-sided heart failure.

Thoracic impedance data showed an inverse relationship with fluid status for a representative patient with combined HF (Figure 2) and with isolated left-sided HF (Figure 3) during hospital admission, also visible by the observed strong correlation coefficients (r_s_>0.700, *P*<.001). The correlation coefficient is higher for patients with isolated left-sided HF.

**Figure 4:**
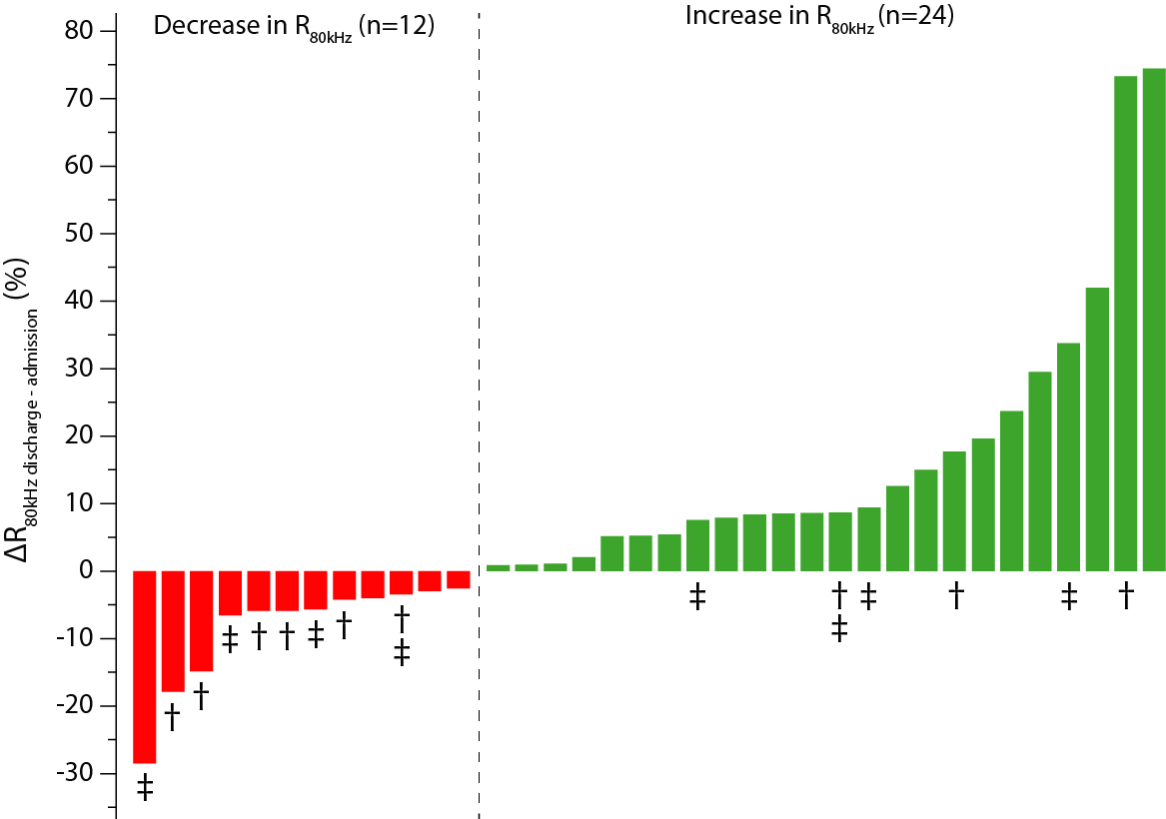
Changes in thoracic impedance at 80 kHz (R_80kHz_) from admission to coronary care unit discharge by patient, including clinical outcome status († all-cause mortality and ‡ hospital admission with a primary diagnosis of heart failure).

For the total sample, a moderate negative correlation was found between changes in fluid balance and relative changes in R_80kHz_ (r_s_= -0.51, *P*<.001). Patients were divided into two groups according to the relative change in R_80kHz_ from admission to coronary care unit discharge: patients with a relative increase in R_80kHz_ and patients with a relative decrease in R_80kHz_. Baseline population characteristics are provided in Table 1. Of the 36 patients, 24 (67%) patients showed a relative increase in R_80kHz_, and 12 (33%) patients showed a relative decrease in R_80kHz_ (Figure 4).

**Table 1:** Comparison of patient baseline characteristics at arrival at the emergency department, grouped according to the relative change in resistance at 80kHz (R_80kHz_) from admission to coronary care unit discharge.

Variables		Patients with decompensated heart failure (n=36)		
		Increase in R_80kHz_ (n=24)	Decrease in R_80kHz_ (n=12)	*P* value
Age (years), mean (SD)		80 (9)	83 (6)	.24
Male sex, n (%)		10 (42)	6 (50)	.64
BMI (kg/m²), mean (SD)		31 (8)	30 (4)	.86
Left ventricular ejection fraction (%)^a^, median (IQR)		55 (39 to 55)	44 (26 to 47)	.057
Heart rate (bpm), mean (SD)		86 (25)	90 (19)	.65
Systolic blood pressure (mm Hg), mean (SD)		144 (23)	147 (33)	.73
Diastolic blood pressure (mm Hg), mean (SD)		74 (18)	72 (26)	.86
Baseline NT-proBNP^b^ (pg/mL)^c^, median (IQR)		3,027 (1681 to 6161)	12,181 (3307 to 17,352)	.052
Total fluid balance during hospitalization (mL), median (IQR)		–3048 (–4396 to 1963)	–1298 (–2225 to 69)	.002
R_80kHz_ at admission (Ω), mean (SD)		42 (20)	46 (18)	.52
R_80kHz_ at coronary care unit discharge (Ω), mean (SD)		48 (22)	44 (18)	.60
Relative R_80kHz_ change from admission to coronary care unit discharge (%), median (IQR)		109 (105 to 122)	94 (85 to 97)	<.001
**Heart failure etiology, n (%)**				
	Ischemic heart disease	10 (42)	4 (33)	.73
	Dilated cardiomyopathy	0 (0)	1 (8)	.33
	Valvular disease	5 (21)	3 (25)	1.00
	Other	9 (38)	4 (33)	.26
**Comorbidities, n (%)**				
	eGFR^d^ <60 mL/min/1.73m²	15 (63)	11 (92)	.12
	Atrial fibrillation	11 (46)	10 (83)	.03
	Implantable electronic cardiac device	5 (21)	3 (25)	.55
	Chronic obstructive pulmonary disease	1 (4)	3 (25)	.10
	Diabetes	7 (29)	6 (50)	.28
**Maintenance therapy, n (%)**				
	Renin-angiotensin system blocker	12 (50)	6 (50)	1.00
	Beta blocker	16 (67)	7 (58)	.72
	(Loop) diuretic	14 (58)	12 (100)	.02

^a^n=31.

^b^NT-proBNP: N-terminal pro-brain natriuretic peptide.

^c^n=26.

^d^eGFR: estimated glomerular filtration rate.

The groups had similar baseline patient characteristics. Significantly fewer patients with atrial fibrillation or undergoing diuretic therapy were present in the group with a relative increase in R_80kHz_. A significant difference in relative R_80kHz_ change from admission to coronary care unit discharge was observed for patients with an increase in R_80kHz_ (109%, IQR 105-122) compared with those with a decrease in R_80kHz_ (94%, IQR 85-97, *P*<.001).

**Figure 5:**
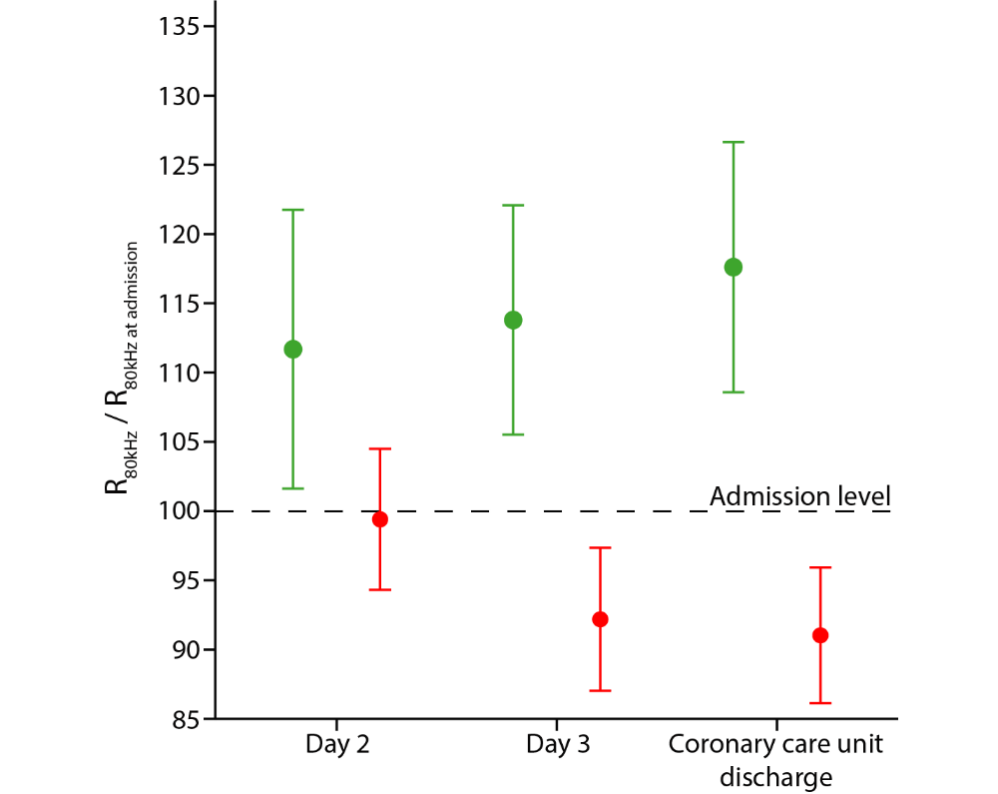
Relative changes in thoracic impedance at 80 kHz (R_80kHz_) from admission to coronary care unit discharge (mean and two times standard error) for patients with a relative increase in R_80kHz_ (green; n=24) or relative decrease in R_80kHz_ (red; n=12).

In the patients with a relative increase in R_80kHz_, the biggest change in R_80kHz_ was observed between the day of admission and the day after admission (+12%; Figure 5). During the subsequent days, smaller relative changes in R_80kHz_ of +2% (between day 2 and day 3) and +4% (between day 3 and the day of coronary care unit discharge) were observed. For patients with a relative decrease in R_80kHz_, smaller relative changes in R_80kHz_ were observed (-0.5%, -7%, and -1%, respectively).

### Clinical Outcome

**Figure 6:**
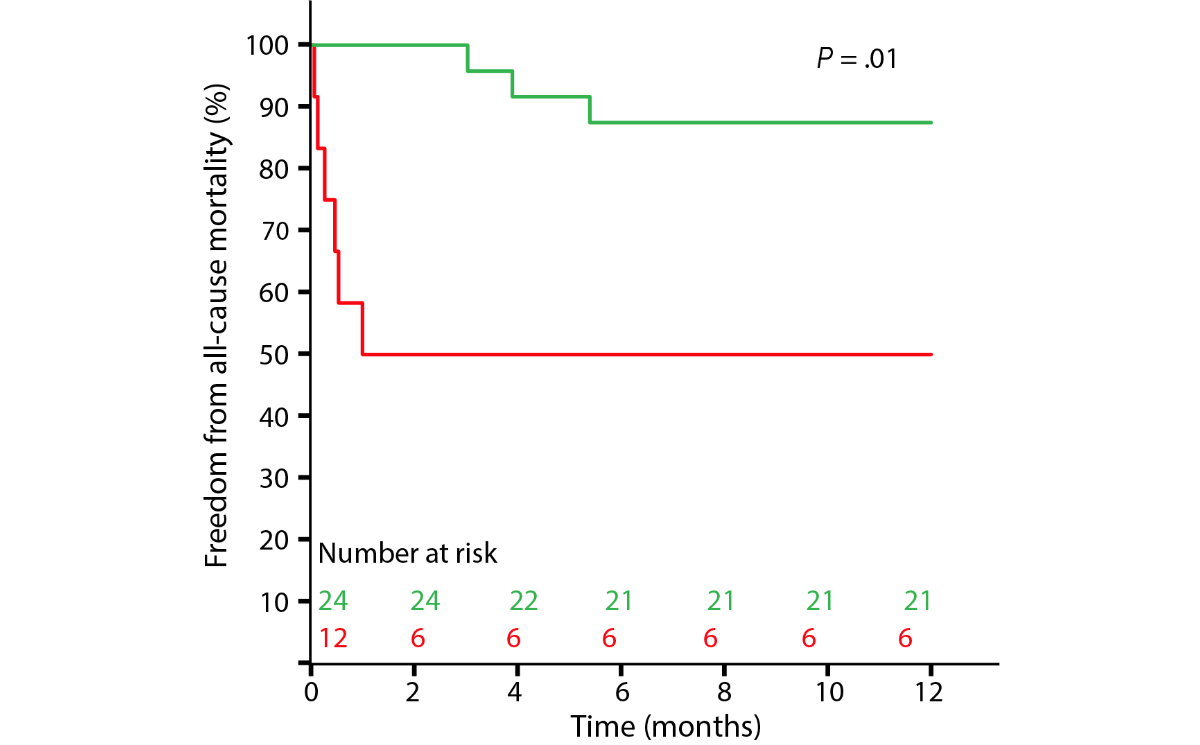
Freedom from all-cause mortality in patients with an increase in R_80kHz_ (green; n=24) versus patients with a decrease in R_80kHz_ (red; n=12).

**Figure 7:**
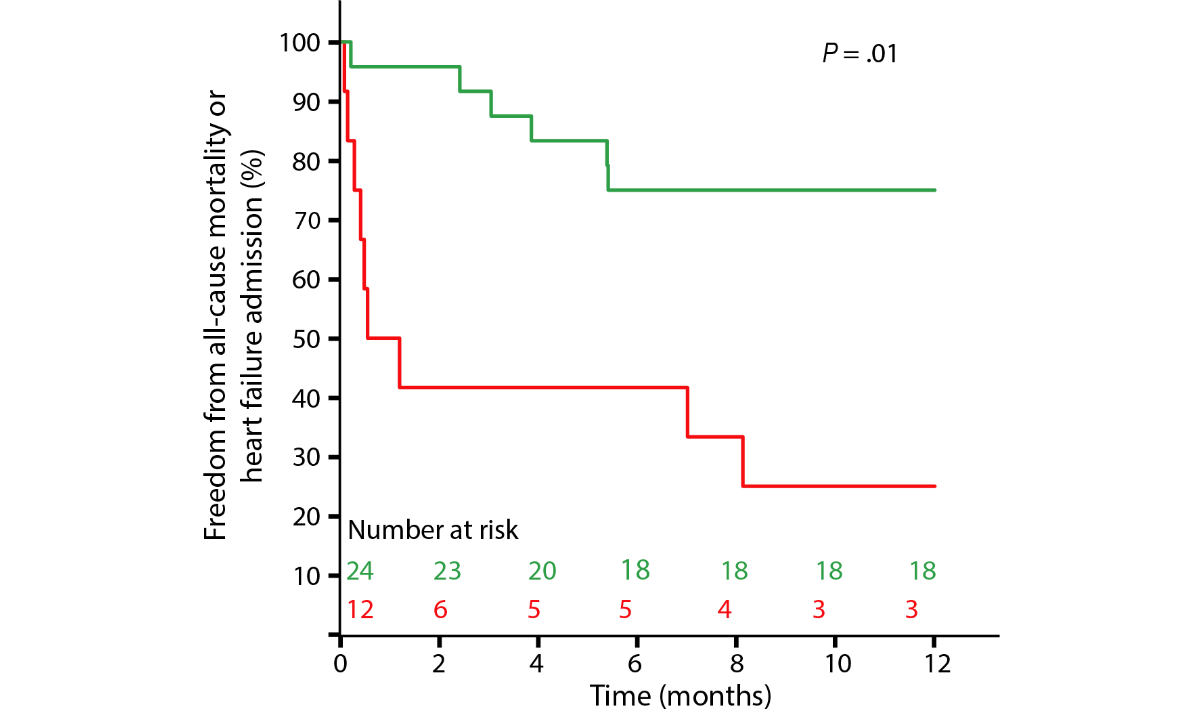
Freedom from all-cause mortality or hospital admission with a primary diagnosis of heart failure for patients with an increase in R_80kHz_ (green; n=24) versus patients with a decrease in R_80kHz_ (red; n=12).

During follow-up, 9 of the 36 patients died, leading to a 1-year survival rate of 75%. Patients with a relative increase in R_80kHz_ had a significantly higher probability of survival (21/24, 88%) than patients with a relative decrease in R_80kHz_ (6/12, 50%, *P*=.01; Figure 6). This difference was already present at 30 days of follow-up (24/24, 100%, and 7/12, 58%, respectively, *P*<.001). After one 1 year of follow-up, 28 of the 36 patients (78%) had not been readmitted to the hospital with a primary diagnosis of HF, and this was not significantly different between patients with a relative increase in R_80kHz_ and patients with a relative decrease in R_80kHz_ (20/24, 83%, and 8/12, 67%, respectively, *P*=.28). At 30 days, these values were 23/24 (96%) and 11/12 (92%), respectively (*P*=.63). Finally, 21 of the 36 (58%) patients survived and had not been readmitted for HF at 1 year of follow-up: 75% of the patients with a relative increase in R_80kHz_ (18/24) and 25% of the patients with a relative decrease in R_80kHz_ (3/12, *P*=.01; Figure 7), compared with 96% (23/24) and 50% (6/12, *P*=.01), respectively, at 30 days of follow-up. Clinical outcome status is included in Figure 4. There were 28 cardiac‑related hospitalizations for 42% (15/36) of the patients. Of the 28 hospitalizations, 27 (96%) were non-elective, and 13 (46%) were HF-related. Clinical outcome results are summarized in Table 2.

**Table 2:** Clinical outcome results at both 30 days and 1 year of follow-up.

Endpoint	30 days of follow-up			1 year of follow-up		
	Increase in R_80kHz_ (n=24)	Decrease in R_80kHz_ (n=12)	*P* value	Increase in R_80kHz_ (n=24)	Decrease in R_80kHz_ (n=12)	*P* value
Freedom from heart failure hospitalization and all-cause mortality, n (%)	23 (96)	6 (50)	.001	18 (75)	3 (25)	.001
Freedom from all-cause mortality, n (%)	24 (100)	7 (58)	<.001	21 (88)	6 (50)	.005
Freedom from heart failure hospitalization, n (%)	23 (96)	11 (92)	.63	20 (83)	8 (67)	.28

Table 3 provides an overview of the Cox regression analysis. A decrease in R_80kHz_ from admission to coronary care unit discharge resulted in a significant hazard ratio of 4.96 (1.82-14.37) for the combined endpoint, mainly driven by all-cause mortality. Multivariate analysis revealed that baseline characteristics that were significantly different between both groups (ie, presence of atrial fibrillation and diuretic use) and clinically relevant parameters (ie, age and left ventricular ejection fraction) had no significant influence on the clinical outcomes (after considering the group variable). Since no factor was significant in the model, the adjusted and unadjusted hazard ratios are the same.

**Table 3:** Cox regression analysis with Firth's penalization for clinical outcome measures.

Endpoint	Hazard ratio	95% CI	*P* value
Heart failure hospitalization and all-cause mortality	4.96	1.82-14.37	.01
All-cause mortality	5.51	1.55-23.32	.02
Heart failure hospitalization	2.10	0.54-8.14	.29

## Discussion

### Principal Findings

Different invasive and non-invasive bioimpedance applications have previously been studied for their potential applications in HF treatment and follow-up [9-13,16,17]. This study provides preliminary evidence about the potential in-hospital use and prognostic value of a wearable thoracic impedance sensor. Individualized bioimpedance monitoring was useful to assess the efficacy of decongestion therapy by tracking changes in a patient’s fluid status. A significant inverse relationship was found between daily fluid balance and thoracic impedance measurements, especially on the individual level. Moreover, patients with an increase in thoracic impedance during initial treatment tended to have a better clinical outcome than patients without an increase in thoracic impedance.

Wearable bioimpedance devices enable longitudinal monitoring and trend analysis in a low-cost, feasible, reproducible, and non-invasive manner. In addition, thoracic impedance changes correlate well with a patient’s fluid balance and could therefore be used to track volume overload [12]. Furthermore, Cuba-Gyllensten et al [13] found the highest correlation between daily fluid levels and thoracic impedance measurements compared to other clinical parameters. Due to the poor prognostic value, serial echocardiographs (eg, left ventricular ejection fraction, E/e’ ratio) or serial biomarkers (eg, pro-BNP, troponins) were not collected as a reference in the current study [4,22-24]. Instead, due to its objective nature, we relied on fluid balance (in and out each hour) as a comparator for fluid status. In this study, the wearable bioimpedance sensor was also inversely related with fluid balance, as expected. Correlations for individual patients were higher than the correlation for the total study population. For the total study sample, a moderate negative correlation was found between changes in fluid balance and relative changes in R_80kHz_ from admission to coronary care unit discharge (r_s_=-0.51, *P*<.001). In a previous study, in which invasive thoracic impedance monitoring was used to target one side of the thorax, the correlation between thoracic impedance and weight changes was –0.65 [11]. Other research on non-invasive bioimpedance monitoring targeting both lungs found even higher correlations [13]. This can be explained by the fact that changes in fluid level occur throughout the entire body, whereas thoracic impedance is a local measurement that, in the current study, only considered the basal part of the left lung. Therefore, when using a non-invasive wearable bioimpedance device, it is very important to consider that the correlation between bioimpedance and fluid balance strongly depends on the location of the excessive fluid and the measurement area of the device. Accordingly, correlations on the individual level can be higher than at the population level, as we demonstrated in our previous study [12]. Similarly, in the present study, a higher correlation coefficient was present for a representative patient with isolated left-sided HF than for a patient with combined HF. In a patient with isolated left-sided HF, most of the extracted fluid originates from the lung area (ie, the measurement location of our wearable device), while in combined HF, fluid is also extracted from the lower peripherals. Measuring a larger area could improve the correlation but limits the possibility of incorporating it in a wearable bioimpedance sensor. It is also important to remember that there are various influencing factors when dealing with non-invasive thoracic impedance measurements. This could further explain the lower correlation for the total study population than for the individual patients in our study. Potential influencing factors for non-invasive bioimpedance measurements include skin conditions, body composition (eg, fat percentage, muscle percentage), food intake, air in the intestines, and pleural cavity fluid. Moreover, external influences can include body posture and electrode placement. Therefore, in our experience, bioimpedance measurements should be interpreted in an individualized longitudinal manner since absolute bioimpedance values exhibit high individual variability. Individually adjusted thresholds and trends, rather than absolute numbers, could help in clinical decision making based on bioimpedance measures.

For patients with an increase in R_80kHz_, the highest change in R_80kHz_ was observed during the first day. This is in accordance with clinical findings from previous research, in which patients with acutely decompensated HF and under diuretic therapy had substantially higher urinary output during the first 24 hours after admission [25]. Interestingly, when compared with patients with a decrease in R_80kHz_, for the patients with an increase in R_80kHz_, a significant survival benefit was observed both for all-cause survival (21/24, 88%, and 6/12, 50%, respectively, *P*=.01) and the composite of all-cause mortality and HF hospitalization (18/24, 75%, and 3/12, 25%, respectively, *P*=.01) at 1 year of follow-up. This difference was already present at 30 days of follow-up. A decrease in R_80kHz_ resulted in a significant hazard ratio of 4.96 (1.82-14.37) for the combined endpoint, and the multivariate analyses, including baseline characteristics that were significantly different between both groups and clinically relevant parameters, revealed no significant influence of these parameters. Thus, the multivariate analysis indicated that the presence of atrial fibrillation, diuretic use, age, and left ventricular ejection fraction did not contribute to the observed differences in clinical outcome between the groups; therefore, this difference was mainly driven by the increase or decrease in thoracic bioimpedance during hospitalization. Effective decongestion, indicated by an increase in thoracic impedance during hospitalization, is thus pivotal for good clinical prognosis. Our wearable bioimpedance monitoring device provides an easy-to-use parameter in this context. The current preliminary results indicate that non-invasive bioimpedance changes early during hospitalization could possibly be used to determine the efficacy of decongestion therapy and improve resource allocation. Accordingly, patients that do not show an improvement in thoracic impedance during the first 48 hours of hospitalization tend to have a poor clinical outcome and require extra attention to optimize decongestion therapy (ie, increase diuretic dose, initiate dialysis therapy). Currently, the efficacy of congestion is mainly determined based on X-ray images or fluid balance information. However, X-ray images are usually only taken once every 24 hours in the acute setting, and although fluid in- and outtake information is recorded every hour, the actual fluid balance is only calculated once every 24 hours. Therefore, bioimpedance could be a more convenient and faster method to determine the efficacy of decongestion therapy. The technique could be easily integrated into existing bedside vital sign monitors for a more continuous recording of fluid status.

Longitudinal invasive hemodynamic monitoring (ie, pressure overload) has already shown its clinical relevance by improving HF management [26]. The proposed wearable bioimpedance monitoring device could be easily integrated in patch form or as textile sensors. It could provide an interesting, non-invasive alternative since it enables longitudinal monitoring of fluid volume in an easy, inexpensive, and comfortable way. Therefore, besides its in-hospital use as an indicator for the efficacy of decongestion therapy or as a prognostic parameter, it could be relevant for in-home monitoring for the early detection of volume overload. Therefore, it could address the increasing burden of worsening HF that requires hospital admission. To ensure patient compliance, a crucial consideration is the autonomous working principle of the wearable form factor, which minimizes patient burden. In the ideal setting, the patient must only attach the wearable device for about 5 minutes daily, during which the device automatically sends the information to the clinical call center. In addition, since the device also enables electrocardiogram data recording and is capable of measuring respiration using bioimpedance, additional parameters can be gathered to obtain a more complete overview of the patient’s health status. In this way, the device could be used to predict upcoming HF decompensation using a multi-parameter approach. However, a new prospective study is needed to investigate its use in an in-home environment.

### Study Limitations

This study should be interpreted in the light of some limitations. Since thoracic impedance measurements were performed on a confined area only covering the basal part of the left lung, changes in bioimpedance measured with the wearable device therefore can only approximate the fluid changes at the whole-body level. However, if both lungs are considered, the device loses the advantages of a comfortable miniaturized wearable device. In our center, the number of patients that are admitted to the coronary care unit for heart decompensation is quite low since we have a multidisciplinary HF clinic in which patients are closely followed. Although the sample size is small, it is comparable in size to other studies that assessed bioimpedance changes in HF patients, and we are convinced that the current findings are strong enough to encourage other research groups to further study the use of non-invasive bioimpedance devices in more detail [11,13,17]. Another limitation is the fact that baseline diuretic use prior to admission at the emergency room was significantly higher in the patients that showed a decrease in thoracic impedance during hospitalization. However, in the clinical outcome analysis, this factor was included in the multivariate analysis and showed no significant influence on the observed differences in clinical outcome. The current results related to clinical outcome are therefore hypothesis-generating, and larger studies are required to support these findings. Finally, serial echocardiographic parameters and biomarkers were not used as reference measures.

### Conclusions

The current study shows that individualized bioimpedance monitoring can be used to track the efficacy of decongestion therapy by measuring changes in fluid status during hospitalization. Changes in fluid balance (in/out) and relative changes in R_80kHz_ from admission to coronary care unit discharge showed a moderate negative correlation on the sample level and higher correlations on the individual level. Early decreases in R_80kHz_ were related with worse clinical outcomes both at 30 days and 1 year of follow-up. Future studies are required to confirm whether bioimpedance monitors could add value in diagnostic evaluation, longitudinal prognostication, therapeutic decision-making, and in-home monitoring for the early detection of volume overload.

## Abbreviations

BNP: brain natriuretic peptide.
eGFR: estimated glomerular filtration rate.
HF: heart failure.
R_80kHz_: resistance at 80 kHz.
